# The Book World of Medicine and Science

**Published:** 1906-07-28

**Authors:** 


### The Book World of Medicine and Science.

Modern Surgical Technique in its Relations to Operations and Wound Treatment. By C. Yelverton Pearson, M.D., M.Ch., F.R.C.S., Professor of Surgery, Queen's College, Cork, etc. Illustrated with two coloured and other plates and one hundred and eleven illustrations in the text. (London: John Bale, Sons, and Daniellson, Limited, Oxford House, 83-91 Great Titchfield Street, Oxford Street, W. Price 12s. 6d.)

The publishers' name is a guarantee of the excellence and practical utility of this volume. Modern surgery has taken many tortuous paths during the last decade, and it is not to be wondered at that knowledge grows grey in those away from centres of education. The general practitioner becomes more and more expert as a physician as time passes and less and less of a surgeon. Practice is wanting to him and he, too often, is painfully aware of his own shortcomings in the light of modern methods involving bacteriology and surgical technique in general. The author had had this in view, and though primarily intended for students, house surgeons, surgical assistants, the work should certainly be in the hands of every general practitioner who aspires to perform surgical operations. The author devotes over 56 pages to general principles and what he calls surgical bacteriology and then takes up the question relating to infection and disinfection. The real work begins with Chapter I. on antiseptics, which is admirably descriptive of the chief antiseptics used in surgery and their actions. The chapter gives the chemical and physiological properties and uses in surgery. The author is so impressed with the importance of this section that he has underlined or italicised a good portion of it as though he were afraid that its importance might be overlooked by his readers. He need have little fear on that score. Everything in the chapter is important and well written and cannot be overlooked by anyone fortunate enough to have this work handy and who desires to have a reason for the surgical faith that is in them. Professor Senn, of Chicago, seems to be a great favourite with the author. He quotes him frequently and uses illustrations from his work on practical surgery. The second part is most practical and deals with prophylactic disinfection, including the hands, the use of gloves and operating costume, preparation of the skin, of the instruments, of the sponges, and of the ligature and suture materials. All these subjects are expounded fully and thoroughly with the skill of a master and not with the ease and accuracy of a practical man. It is astonishing how much one has to learn concerning the apparently simple process of preparing a ligature for operation purposes. The nature and mode of sterilisation of absorbent dressings is always a fascinating subject, and Dr. Pearson makes it still more so. The sterilisation of absorbent dressings leads to a description of the materials which are required to render them sterile and an account of the best of them is given. The third part of the work on wound technique deals with the nature of wounds and the prevention and arrest of hæmorrhage and gives an account of the different ligatures—namely, the granny, the reef, the surgeon's, the continuous ligature, the Staffordshire knot, the interlocked ligature, and other methods of arresting hæmorrhage. A chapter on needles is important. It is a pity he does not tell us how to prevent needles from rusting. He says, however, rusting is less likely to occur when the needles are carried in a flannel book than in metallic boxes. The different sutures are then dealt with and beautifully illustrated, and then the questions of drainage and the different forms of tubes are considered. The operative technique is the last section of the work and presents a complete guide to all that is modern and safe.

Muscles and Nerves; an Atlas of the Superficial Muscles and the Principal Motor Nerves of the Human Body, for the Use of Students of Anatomy and Nurses. By Louis B. Rawling, F.R.C.S., Assistant Surgeon and Senior Demonstrator of Anatomy at St. Bartholomew's Hospital. (London: The Scientific Press, Ltd., 28 and 29 Southampton Street, Strand, W.C. 1906. Price 3s. 6d.)

The author is well known for the excellence of his published works on topographical and surface anatomy. A knowledge of the superficial markings of the human body cannot be too deeply impressed upon all who in any capacity undertake treatment of disease. If this knowledge is essential to the proper exercise of the medical profession, it is no less so in those who are required to render first aid in ambulance work or to nurse the patient either at home or in hospital. This little atlas will be most useful also to those engaged in teaching physical exercises, drill, the Swedish movements, or any form of massage. Indeed, these different forms of treatment cannot be practised with any advantage without at least a complete knowledge of the superficial markings of the body. The recent revival in physical culture and gymnastics render an atlas of this kind of great practical utility. The plan adopted in the work has been to photograph a well-developed male in positions best fitted to show the surface markings, and then to give a plan of the superficial muscles shaded according to the direction of their fibres, and to mark out those nerves which from their exposed position are prone to injury or disease. There are four plates representing: I. Front View; II. the Posterior View; III. the Lateral Aspect; IV. A kind of reclining posture showing the inner aspect of the arm and the thigh. The Atlas is well got up and an examination of the figures will give the student an insight into the superficial muscular formation of the human body and an idea of the position of the nerves, as well as prove an adequate guide to the more important landmarks. With this in view the Atlas has our confident recommendation.

German Grammar for Science Students. By W. A. Osborne, M.B., D.Sc., Professor of Physiology and Histology in the University of Melbourne, and Ethel E. Osborne, M.Sc. (London: Whittaker and Company, 2 White Hart Street, Paternoster Square, E.C. New York: 64 and 65 Fifth Avenue. 1906. Pp. 106. Price 2s. 6d.)

Professor Osler's advice to the medical students of St. Thomas's Hospital to acquire a knowledge of modern languages, especially of German, has called attention to the manner in which science is beholden to German literature. Advanced students of medicine find this acquirement more and more essential to their success, which depends in large measure on an adequate study of German scientific methods and research. This little German Grammar embodies a happy means of hitching up the early school-day study of German and in a few short pages bringing it back to a happy and practical acquaintance with the language of the laboratory in German instead of English. All medical students who value the advice of Professor Osler and intend to become sufficiently acquainted with the German language to enable them with the aid of a good dictionary to read with some fluency the books and journals published in the German tongue, should not fail to provide themselves with Osborne's German Grammar. Any one in real earnest should be able to master its contents during a month's holiday and be thereby enabled to derive much pleasure and profit from the German scientific magazines.

